# Supplementary material for: Free-oscillation technique: The effect of the magnitude of the impulse applied on muscle and tendon stiffness around the ankle
Source: PLoS One. 2023 Jun 16;18(6):e0286847. doi: 10.1371/journal.pone.0286847 (PMC10275422; doi:10.1371/journal.pone.0286847)
Supplement: Attachment — Submitted filename: PONE-D-23-03008_reviewer.docx [file pone.0286847.s001.docx]

Summary:

Impulse loads were applied to the foot of healthy individuals to assess the stiffness of the muscles and tendons surrounding the ankle joint. From these trails, mathematical equations were used to determine the stiffness of the muscles and respective tendons surrounding the ankle joint.

Comments:

Abstract: the references to certain abbreviations (Mdn, and KN/m/KN) are not provided, nor are the terms “apparent” stiffness and “true” stiffness well defined

Introduction:

- A reference to the definition of tissue stiffness is needed in the first paragraph
- The purpose is provided, but there is a lack of a hypothesis to drive the research agenda of this project

Methods:

- The authors reference the Faria et al study for the assessment of impulse, but they need to provide additional information to allow the readers in determining how the forces were recorded and measured
- The stiffness assessment is poorly explained. It is suggested that the authors use a diagram to illustrate the procedure for testing the stiffness
- How many trials of the impulse loading was applied, and were these loads randomized to reduce a potential order effect?
- It is also not clear if sufficient rest periods were provided between trails/loads – this is important as repeated loading will modify the stiffness of the tissues
- Since stiffness is the measure that is of most concern, the authors mention ankle joint stiffness and then stiffness of the muscles and tendons around the ankle joint. Which is the stiffness you are truly assessing? Furthermore, were specific muscles assessed, such as the plantar flexors, or was this analysis inclusive of plantar flexor and dorsi flexor muscles?

Results:

- Paragraph 1 does not communicate to the reader well what was reported from the methods. This may be due to the lack of definitions to the abbreviations (although these abbreviations are given in the footer of the table that follows – Table 1)
- Further, the lack of experimental explanation that is expected in the Methods section makes it difficult to clearly interpret the results

Discussion:

- The interpretation of the data, based upon the presented results, should be presented cautiously as many details are not communicated well in the previous sections
- While the theoretical argument brought forward by the authors regarding the stiffness of the muscle versus the tendon is plausible, the data are not as clear – again this is due to a lack of clarity in the Methods section
- What were the limitations of this study? The authors have stated the underlying concepts, but what limits were present that may have influenced the results?
